# Skill complementarity enhances heterophily in collaboration networks

**DOI:** 10.1038/srep18727

**Published:** 2016-01-08

**Authors:** Wen-Jie Xie, Ming-Xia Li, Zhi-Qiang Jiang, Qun-Zhao Tan, Boris Podobnik, Wei-Xing Zhou, H. Eugene Stanley

**Affiliations:** 1 School of Business, East China University of Science and Technology, Shanghai 200237, China; 2 Postdoctoral Research Station, East China University of Science and Technology, Shanghai 200237, China; 3 Departmenent of Mathematics, East China University of Science and Technology, Shanghai 200237, China; 4 Research Center for Econophysics, East China University of Science and Technology, Shanghai 200237, China; 5 Shanda Games Ltd., 690 Bibo Road, Shanghai 201203, China; 6 Center for Polymer Studies and Department of Physics, Boston University, Boston, MA 02215, USA; 7 Zagreb School of Economics and Management, 10000 Zagreb, Croatia; 8 Luxembourg School of Business, Luxembourg; 9 Faculty of Civil Engineering, University of Rijeka, 51000 Rijeka, Croatia; 10 Faculty of Economics, University of Ljubljana, 1000 Ljubljana, Slovenia

## Abstract

Much empirical evidence shows that individuals usually exhibit significant homophily in social networks. We demonstrate, however, skill complementarity enhances heterophily in the formation of collaboration networks, where people prefer to forge social ties with people who have professions different from their own. We construct a model to quantify the heterophily by assuming that individuals choose collaborators to maximize utility. Using a huge database of online societies, we find evidence of heterophily in collaboration networks. The results of model calibration confirm the presence of heterophily. Both empirical analysis and model calibration show that the heterophilous feature is persistent along the evolution of online societies. Furthermore, the degree of skill complementarity is positively correlated with their production output. Our work sheds new light on the scientific research utility of virtual worlds for studying human behaviors in complex socioeconomic systems.

Complexity emerges in the evolving and self-organizing processes of many natural, social, technological, and biological systems. The constituents of a complex system interact with each other and form complex evolving networks, where the constituents are nodes and their interaction relationships are links^1^,^2^,^3^,^4^,^5^,^6^. For many real networks, the link formation process follows either the global principle of popularity in which a node tends to link with high-degree nodes^7^,^8^, or the local principle of similarity in which a node tends to link with nodes having traits similar to its own^9^, or a tradeoff between them^9^.

In the sociological literature the local principle of similarity, i.e., the phenomenon that “birds of a feather flock together,” is known as homophily^10^. There is much empirical evidence indicating that individuals prefer to forge social ties with people whose traits such as education, race, age, and sex are the same as their own^11^,^12^,^13^,^14^. Such homophilous behaviors are ubiquitous in social networks and have been well documented^10^,^11^,^12^,^14^,^15^,^16^,^17^,^18^. In addition, the similarity shared by individuals in a group is often a significant predictor of a group’s altruism level and its ability to cooperate^19^. Sociological literature argues that human societies tend to display two social systems: (i) homophilous, in which people seek out people who are similar, and (ii) heterophilous, in which people seek out people who are different^20^. The evidence indicating the actual existence of heterophilous societies is rare, however. One example is to study team formation processes in offline gangs and online games depending on the heterogeneity of agents’ attributes^21^.

In general, it has long been accepted that one of the most significant factors in increasing productivity in modern human societies has been the division of labor^22^. Thus we might assume that people in modern societies now prefer to forge links or collaborate with those who have complementary productive skills and that socioeconomic networks are becoming increasingly heterophilous, but no direct evidence of this has been documented. The availability of big data recorded from massively multiplayer online role-playing games (MMORPGs) enables us to test social and economic hypotheses and theories—such as this one—in large-scale virtual populations^23^ and gain a deeper understanding of our social and economic behaviors^24^,^25^,^26^,^27^,^28^,^29^,^30^,^31^,^32^.

In this work, we study the collaboration formation process of individuals with different professional skills. A mathematical model is proposed by assuming that individuals in socioeconomic systems choose collaborators that are of maximum utility. Based on the evolving collaboration networks of 124 virtual worlds in which the agents (virtual people) belong to three different professions possessing different skills, empirical analysis and model calibration unveil that the agents prefer to collaborate with others of different professions. We further construct two measures to quantify the degree of complementarity of virtual societies. We find that social complementarity positively correlates with economic output.

## Results

### A model of collaboration formation

Consider a society or a community *s* on day *t*, whose size *N*_*s*_ is the number of *s*-agents. The number of (*s*, *i*)-agents is denoted by *N*_*s*,*i*_, where *i* = 1, 2, and 3 stand for the three professions. Hence 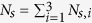. The ratio of *i*-agents in society *s* is


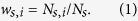


The average number of *j*-collaborators of an (*s*, *i*)-agent is *f*_*s*,*ij*_. Hence, the average number of collaborators that an (*s*, *i*)-agent has is 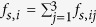. The average proportion of *j*-collaborators in all collaborators of an *i*-agent is


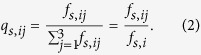


Note that *q*_*s*,*ii*_ is the homophily index^15^,^33^. If *i*-agents have zero preference for collaborating with *j*-agents, we have *q*_*s*,*ij*_ = *w*_*s*,*j*_. If *i*-agents prefer to collaborate with *j*-agents, we have *q*_*s*,*ij*_ > *w*_*s*,*j*_. In this case the *i*-agents are homophilous when *j* = *i* and the *i*-agents are heterophilous when *j* ≠ *i*.

An agent seeks collaborators when she/he finds it difficult to complete a task alone. If there is no collaboration preference, the proportion of (*s*, *j*)-collaborators that an (*s*, *i*)-agent has is identical to the proportion of *j*-agents in the group, that is *q*_*s*,*ij*_ = *w*_*s*,*j*_. Hence the number of (*s*, *j*)-collaborators of an (*s*, *i*)-agent is 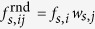. However, in a society with a division of labor, the choice of collaborators has a significant influence on the completion of the task and it is better to have collaborators with complementary skills. Therefore, the number and skill configuration (or distribution) of an agent’s collaborators are the main determinants of her utility. We assume that, for an (*s*, *i*)-agent, there is an optimal configuration of collaborators with different skills, 
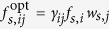
, where the preference coefficients *γ*_*ij*_ are independent of society *s*. If the skill configuration in the collaborator list of an agent is optimal, her utility reaches its maximum. If the skill configuration deviates from that optimal value, her utility is reduced. In other words, the utility of an (*s*, *i*)-agent increases when her/his real number *f*_*s*,*ij*_ of (*s*, *j*)-collaborators approaches the optimal value 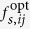 and reaches its maximum 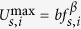 when her/his collaborator configuration is optimal such that 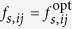. According to the law of diminishing marginal utility, we have *β* < 1. Therefore, the utility function of an (*s*, *i*)-agent is


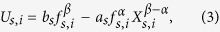


where


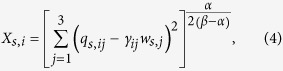


in which *γ*_*ij*_ is the preference of (*s*, *i*)-agents for (*s*, *j*)-agents and *α* > 0 since the second term in Eq. (3) quantifies the amount of utility decrease that is proportional to the deviation of the real configuration to the optimal configuration. If *i*-agents do not have any preference on *j*-agents such that *q*_*s*,*ij*_ = *w*_*s*,*j*_ for all societies, we have *γ*_*ij*_ = 1. If *i*-agents prefer *j*-agents, we have *γ*_*ij*_ > 1. If *i*-agents prefer not to collaborate with *j*-agents, we have *γ*_*ij*_ < 1. For {*i*, *j*, *k*} = {1, 2, 3}, if *γ*_*ij*_ > *γ*_*ik*_, then *i*-agents prefer *j*-agents over *k*-agents. To maintain a collaboration network of size *f*_*s*,*i*_, the (*s*, *i*)-agent suffers a cost proportional to *f*_*s*,*i*_ 12,


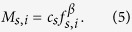


According to the above model, the overall utility in the decision-making process is


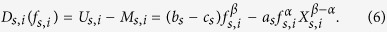


By maximizing *D*_*s*,*i*_(*f*_*s*,*i*_), we can estimate the parameters *γ*_*ij*_ (see *Materials and Methods*).

### Empirical analysis

**Figure 1:**
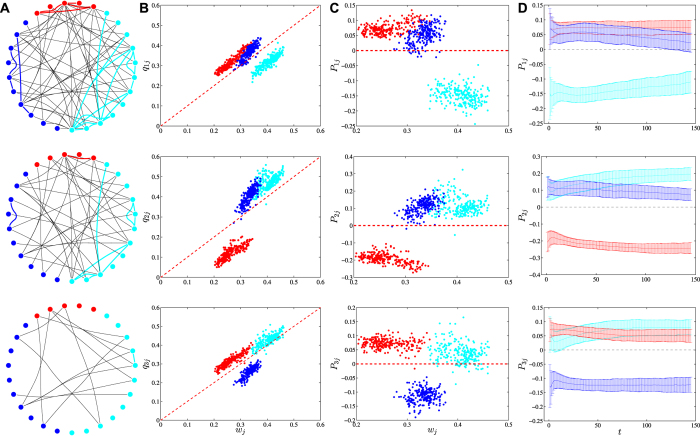
Empirical evidence of heterophily in the socioeconomic networks of virtual societies on a typical day *t* = 15. Warriors, priests and mages are marked respectively in cyan, red and blue. (**A**) Networks of 27 agents randomly chosen from a virtual society filtered by three intimacy thresholds *I*_*c*_ = 0, 100 and 2000 (top to bottom). (**B**) Dependence of *q*_*s*,*ij*_ on relative size *w*_*s*,*j*_ for all virtual societies for *I*_*c*_ = 100. In each plot, there are three well isolated clusters. For most societies, *q*_*s*,*ij*_ > *w*_*s*,*j*_ when *i* ≠ *j* and *q*_*s*,*ij*_ < *w*_*s*,*j*_ when *i* = *j*. (**C**) Dependence of preference measure *P*_*s*,*ij*_ on relative size *w*_*s*,*j*_ for all societies for *I*_*c*_ = 100. There are also three well separated clusters in each plot. For most societies, *P*_*s*,*ij*_ > 0 when *i* ≠ *j* and *P*_*s*,*ij*_ < 0 when *i* = *j*. (**D**) Evolution of the averaged preference measure *P*_*s*,*ij*_ over all virtual societies for *I*_*c*_ = 100. The preference measures are roughly persistent.

Figure 1A shows the collaboration networks on day *t* = 15 of a group of 27 agents randomly chosen from a virtual society filtered by three intimacy thresholds *I*_*c*_ = 0, 100, and 2000. There are 12 warriors, 5 priests, and 10 mages. If *i*-agents are homophilous (neutral, heterophilous) in their collaboration-forging process, the proportion of links between *i*-agents is greater than (equal to, less than) the square of the proportion of *i*-agents (0.1975 for warriors, 0.0343 for priests, and 0.1372 for mages). For *I*_*c*_ = 0, there are 77 links including 15 intra-warrior links, 4 intra-priest links, and 4 intra-mage links. The proportions of intra-profession links are 0.1948 for warriors, 0.0519 for priests, and 0.0519 for mages. For *I*_*c*_ = 100, there are 48 links including 8 intra-warrior links, 1 intra-priest link, and 1 intra-mage link. The proportions of intra-profession links are 0.1667 for warriors, 0.0208 for priests, and 0.0208 for mages. For *I*_*c*_ = 2000, there are 15 links including only one intra-warrior link and no intra-priest and intra-mage links. The proportions of intra-profession links are 0.0667 for warriors and 0 for priests and mages. Hence, the agents in Fig. 1A are heterophious except for priests when *I*_*c*_ = 0. We will show below that heterophily is not a specific characteristic for these 27 agents but a universal feature presents in all the virtual societies.

Figure 1B shows that when *t* = 15 and *I*_*c*_ = 100 most virtual societies have *q*_*s*,*ij*_ > *w*_*s*,*j*_ when *i* ≠ *j*, but *q*_*s*,*ij*_ < *w*_*s*,*j*_ when *i* = *j*. Such heterophilous patterns are observed for other values of *t* and *I*_*c*_ as well (see Fig. S1).

Similar to the inbreeding homophily index^15^,^33^, we define the collaboration preference index to be


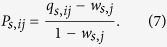


Note that *P*_*s*,*ii*_ is the inbreeding homophily value^15^,^33^. If *i*-agents have no preference to collaborate with *j*-agents, we have *P*_*s*,*ij*_ = 0. If *i*-agents prefer to collaborate with *j*-agents, we have *P*_*s*,*ij*_ > 0. In the latter case, the *i*-agents are homophilous when *j* = *i* and heterophilous when *j* ≠ *i*. Empirical results show that for most virtual societies *P*_*s*,*ij*_ > 0 when *i* ≠ *j*, but *P*_*s*,*ij*_ < 0 when *i* = *j* (Fig. 1C and Fig. S2). Thus in socioeconomic networks the agents are heterophilous.

Figure 1D shows the evolution of preference values *P*_*ij*_ averaged over all societies on the same day for *I*_*c*_ = 100. Although these curves exhibit mild trends, it is evident that the heterophilous feature is persistent as the virtual societies develop (see also Fig. S3).

### Quantifying collaboration preference

**Figure 2:**
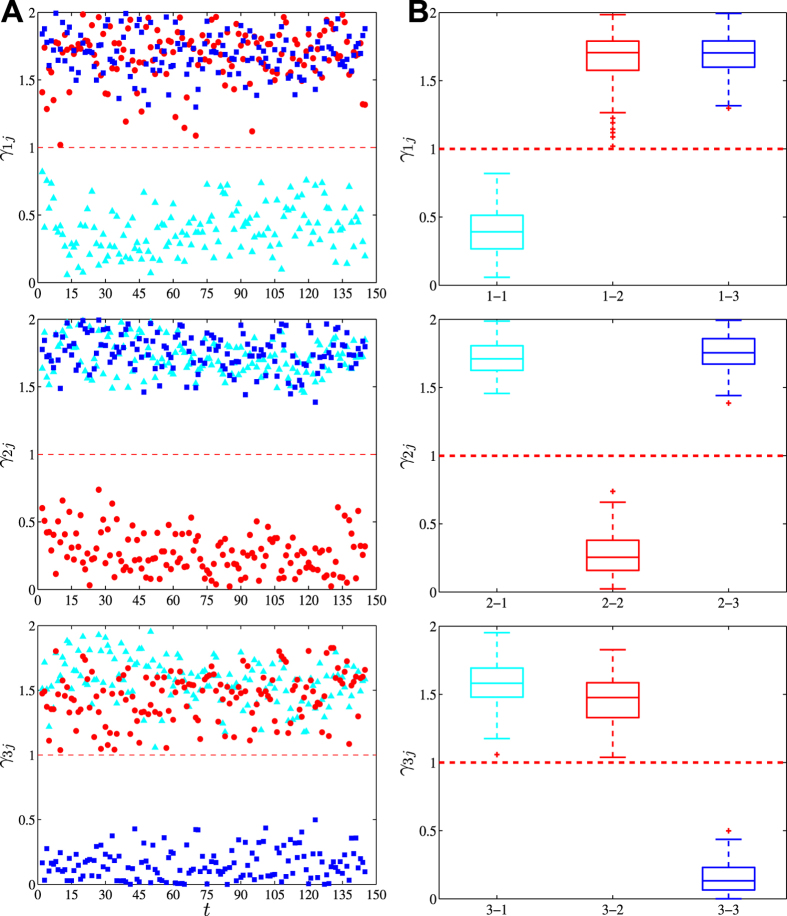
Preference coefficients *γ*_*ij*_ for socioeconomic networks with the intimacy threshold being *I*_*c*_ = 100. (**A**) Daily evolution of the nine preference coefficients *γ*_*ij*_ with 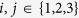. The color of a point (*t*, *γ*_*ij*_) is determined by *j*: cyan, red and blue for *j* = 1, 2 and 3, respectively. The nine points for a given *t* were determined simultaneously in one calibration. (**B**) Box plots of *γ*_*ij*_ shown in (**A**).

To calibrate the model, we follow and further develop the econometric method presented in ref. 12 (see *Materials and Methods*). We obtain the values of *γ*_*ij*_ for each intimacy threshold *I*_*c*_ on each day *t*. Figure 2A shows the evolution of preference coefficients *γ*_*ij*_ for socioeconomic networks using the intimacy threshold *I*_*c*_ = 100, and Fig. 2B shows the average preference coefficients over all days. More results are given in Fig. S4 and Fig. S5 for *I*_*c*_ = 0, 1, 10, 500, 1000, and 2000. The *F*-tests presented in *Materials and Methods* show that all the results are significant at the 0.1% level (see *SI Tables*).

All the estimated values of the *γ*_*ii*_ coefficients are less than 1, while all the *γ*_*ij*_ values for *i* ≠ *j* are greater than 1. This indicates that the agents are not seeking same-profession agents but different-profession agents and are thus heterophilous. In most cases, especially when the intimacy threshold *I*_*c*_ is not large, the *γ*_*ij*_(*I*_*c*_, *t*) values do not have a trend along the evolution of virtual worlds. When *I*_*c*_ is large, however, we observe an increasing trend in *γ*_13_(*I*_*c*_, *t*) for *I*_*c*_ = 1000 and 2000, in *γ*_23_(*I*_*c*_, *t*) for *I*_*c*_ = 1000 and 2000, and in *γ*_32_(*I*_*c*_, *t*) for *I*_*c*_ = 500, 1000 and 2000 (Fig. S4). We find that the preference coefficients might change with the increase of *I*_*c*_ (Fig. S4 and Fig. S5). For warriors, *γ*_11_ and *γ*_13_ decreases, while *γ*_12_ increases. For priests, *γ*_21_ increases, *γ*_22_ does not exhibit evident trend, while *γ*_23_ decreases. For warriors, *γ*_31_ increases, *γ*_32_ decreases, while *γ*_12_ increases for large *I*_*c*_ values.

There are also intriguing patterns of relative collaboration preference as quantified by *γ*_*ij*_ − *γ*_*ik*_ where *i*, *j* and *k* correspond to the three professions (Fig. 2B and Fig. S5). On average, warriors prefer priests over mages and this relative preference enhances when *I*_*c*_ becomes greater but reduces slightly when *t* increases for large *I*_*c*_ values. Priests prefer mages over warriors when *I*_*c*_ values are small and prefer warriors over mages when *I*_*c*_ values are large. For large *I*_*c*_, priests’ relative preference on warriors over mages decreases along time *t*. Mages prefer priests over warriors when *I*_*c*_ is small and prefers warriors over priests when *I*_*c*_ is large. For large *I*_*c*_, mages’ relative preference on warriors over priests also decreases along time *t*.

### Group complementarity and economic output

To measure the economic implications of heterophilous preference in socioeconomic networks, we investigate the relationship between complementarity of professions and economic performance. Consider the socioeconomic network 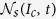 of a virtual society with intimacy threshold *I*_*c*_ on day *t*. Economic production utilizes virtual money and goods that are converted to a standardized currency (see *Materials and Methods*). For each member agent *a* in 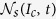, we calculate her production output in the week from *t* − 6 to *t*, denoted as *Y*_*s*,*a*_(*t*). The economic performance of the agents in 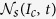 is defined as the output per capita,


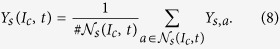


One measure of profession complementarity can be defined as the sum of preference measures between the three types of agents,


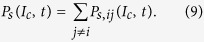


Alternatively, we can measure complementarity by determining how much the real collaborator configuration *q*_*s*,*ij*_ deviates from the optimal collaborator configuration *γ*_*ij*_*w*_*s*,*j*_ (see *Materials and Methods*). The lower the deviation, the higher the degree of complementarity. Thus, we have


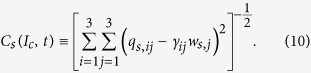


**Figure 3:**
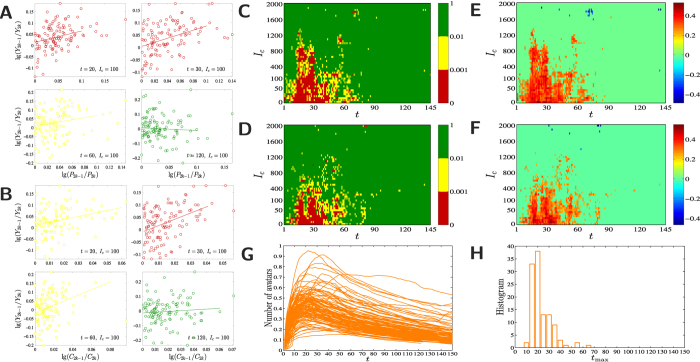
Relation between complementarity of collaboration network and economic output. (**A**) Examples of correlations between lg(*P*_2*k*−1_/*P*_2*k*_) and lg(*Y*_2*k*−1_/*Y*_2*k*_). (**B**) Examples of correlations between lg(*C*_2*k*−1_/*C*_2*k*_) and lg(*Y*_2*k*−1_/*Y*_2*k*_). (**C**) The *p*-value of the correlation between lg(*P*_2*k*−1_/*P*_2*k*_) and lg(*Y*_2*k*−1_/*Y*_2*k*_) for different values of *I*_*c*_ and *t* (in units of days). A give grid (*t*, *I*_*c*_) is colored as red or yellow if the correlation is significant at the 0.001 level or the 0.01 level. Otherwise, the grid is colored as green. (**D**) The *p*-value of the correlation between lg(*C*_2*k*−1_/*C*_2*k*_) and lg(*Y*_2*k*−1_/*Y*_2*k*_). (**E**) Correlation coefficient *ρ* between lg(*P*_2*k*−1_/*P*_2*k*_) and lg(*Y*_2*k*−1_/*Y*_2*k*_) for different values of *I*_*c*_ and *t*. The correlation coefficient is set to be zero is the correlation is insignificant at the 0.01 level. (**F**) Correlation coefficient *ρ* between lg(*C*_2*k*−1_/*C*_2*k*_) and lg(*Y*_2*k*−1_/*Y*_2*k*_). (**G**) Evolution of the number of active agents in different virtual worlds. (**H**) Histogram of *t*_max_ which is the date that a virtual world has historically the maximum active agents.

To make these results comparable for different virtual worlds, we investigate the relative quantities between two societies in the same world, lg(*P*_2*k*−1_/*P*_2*k*_), lg(*C*_2*k*−1_/*C*_2*k*_) and lg(*Y*_2*k*−1_/*Y*_2*k*_), rather than focusing on each society separately. Both measures of complementarity correlate strongly with the relative economic output when *t* and *I* are not large (Fig. 3A–F, Fig. S7, and Fig. S8). For the first few days (small *t*), most agents strive to achieve higher levels by implementing specific tasks with small economic outputs. Other agents attempt to obtain high intimacy levels by killing monsters in locations unrelated to economic outputs. In both cases the agents intend to form complementary collaboration networks, but their activities are not focused on economic outputs. With the development of a virtual world, the number of active agents increases and reaches a maximum at time *t*_max_ and then decays (Fig. 3G). When the activity level of a virtual world decreases, the intent of the agents moves away from production and the collaboration structure is increasingly unrelated to economic activities. This is consistent with the fact that the spectrum of *t*_max_ has a distribution similar to the significant correlations between complementarity and economic output (Fig. 3H).

## Discussion

Overwhelming empirical evidence has shown that most social networks are homophilous. The probability that two nodes will connect is higher if they share similar traits. Our analysis of virtual worlds in which division of labor is operative demonstrates the important role of complementarity. In those socioeconomic networks individuals have the motivation to cooperate, and in the formation of the network individuals exhibit a heterophilous preference for those with complementary productive skills. Although mapping human behavior in virtual worlds to real-world human behavior is a subtle process^34^, we believe that they share an intrinsic commonality because agents in virtual worlds are, in fact, controlled by real-world people. In particular, agents consciously form teams to accomplish tasks more successfully and effectively. More generally, growing evidence shows significant similarities in the behaviors of online agents and real-world humans^23^,^35^,^36^,^37^,^38^,^39^,^40^,^41^,^42^,^43^.

In reality, human’s preference is multidimensional in their traits^13^. The situation in virtual societies is a little different. Indeed, the way people interact with each other has significantly changed from the old days, particularly due to the impact of the Internet. In the modern time, people can meet through the Internet in the virtual world instead of physically getting together to dine, drink, and talk to forge ties. Personal traits become less important in virtual societies while agents’ profession skill is identified as a dominating trait in virtual societies. Like most MMORPGs, the system is set up in a way that a party requires different roles to function optimally. In this sense, the main result of the paper would primarily reflect the design decisions of the game developers. On the other hand, however, such a setup is trying to mimic the real human society, in which people have different and diverse skills and hence there appears the division of labor^22^. Hence, the results documented in this work have a general significance.

The economic model proposed in this work is different from the one in ref. 12. The essential difference is in the assumption of the utility function. The choice of the utility function may have significant impact on the outcome of the model. We calibrated the original model in ref. 12 and the estimates of parameters suggested a homophilous behavior, which is inconsistent with the empirical results presented in Fig. 1. Also, we have used a modified method of model calibration. Moreover, our model allows us to determine not only if *i*-agents are homophilous or heterophilous but also the preference of one type of agents to any other type of agents. Hence, our model is more general and can be applied to other systems.

The relationship between social networks and economic output has been studied previously. It has been found, for example, that the diversity of individual relationships within a community strongly correlates with the economic development of the community^44^ and is directly associated with higher productivity for both individuals and the community^45^,^46^. Because, to date, detailed real data at the population level of societies have been unavailable, this correspondence between professional skill and economic performance has not been quantified. Here we have begun to fill this data gap and also to highlight the usefulness of virtual worlds in carrying out research in economics and sociology^23^. One potential implication of our findings is that if a team leader or a firm manager recruits new members according the complementarity of their skills, the team’s productivity will increase and the firm’s economic well-being grow.

## Materials and Methods

### Data description

We use a huge database recorded from *K* = 124 servers of a popular MMORPG in China to uncover the patterns characterizing virtual socioeconomic networks. In a virtual world residing in a server there are two opposing camps or societies. Two agents can choose collaborators, and a measure of closeness called intimacy is assigned to the collaboration link. When two collaborators in the same society collaborate to accomplish a task, their intimacy level increases. Two agents from different societies can also collaborate, but their intimacy level remains zero. Hence the social networks of the two camps are essentially separate. We can regard the two camps as two societies, thus giving us *S* = 248 virtual societies. For convenience, *s* = 2*k* − 1 and *s* = 2*k* stand respectively for the two societies in the same virtual world *k*. Two agents are defined as collaborators if they both are on the collaborator list and their intimacy exceeds *I*_*c*_. We consider many temporal collaboration networks. On day *t* in a virtual society *s*, a network 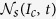 is a network in which the intimacies of all edges are no less than a threshold *I*_*c*_, which can be disconnected (Fig. 1A).

There are a lot of different types of tasks in the virtual societies, which are embedded for agents of all levels. In some levels, the system will ask the agents to kill given amounts of different types of monsters. In other levels, the agents are asked to deliver something to a specific NPC (not-a-person character). And so on so forth. These tasks are usually not easy for the associated agents. However, they can ask their collaborators for help to form a team and fulfill the tasks together. Agents can also form teams to kill monsters and make productions. All these collaborations will increase the intimacy of the collaborating agents in the same team.

In each society there are three professions (warrior, priest, and mage). We use subscripts 1, 2, and 3 to stand respectively for the three professions: warrior, priest and mage. For simplicity, we define several notations as follows. An *s***-agent** is an agent belonging to society *s*. An *i***-agent** is an agent having profession *i*. Similarly, an *i***-collaborator** is a collaborator having profession *i*. An (*s*, *i*)**-agent** or (*s*, *i*)**-collaborator** is an *i*-agent or *i*-collaborator in society *s*.

### Model calibration

An (*s*, *i*)-agent solves the following decision-making problem of how many collaborators to have


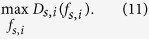


It follows that


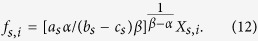


Note that the *γ*_*ij*_ values are affected only by the professions and remain the same for different societies. This enables us to estimate the parameters.

The solution (12) denotes the average behavior (decision) of all agents having the same profession in a given society. If we consider an arbitrary agent *a*, we must add a noise term^12^,


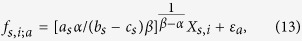


which means that the “realized” number of collaborators agent *a* has is the sum of a universal (or systemic) term and an idiosyncratic error term. The error term is assumed to have mean 0 and variance *σ*^2^. Note that this assumption states that the variance of any agent of any profession is the same.

We denote *N*_*s*_ as the size of society *s* and *w*_*s*,*i*_ as the fraction of *i*-agents in society *s*. Hence the number of *i*-agents in society *s* is *N*_*s*_*w*_*s*,*i*_, and the expectation of the aggregated number of collaborators that *i*-agents have in society *s* is *N*_*s*_*w*_*s*,*i*_*f*_*s*,*i*_. According to Eq. (13), we have


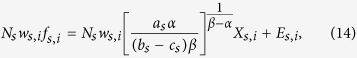


where *E*_*s*,*i*_ has mean 0 and variance (*N*_*s*_*w*_*s*,*i*_*σ*)^2^ = (*N*_*s*_*w*_*s*,*i*_)^2^*σ*^2^.

It follows that, for *i* ≠ *j*,


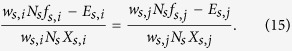


Following ref. 12, we obtain an error for society *s*:


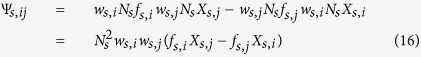



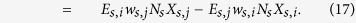


According to Eq. (17), we find that the mean of Ψ_*s*,*i*,*j*_ is 0 and the variance is *ϕ*_*s*,*ij*_*σ*^2^, where


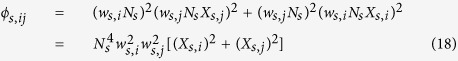


Thus the normalized variable 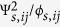 has mean 0 and variance *σ*^2^ for any society *s*. The sum of squared errors 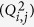 over all societies in the sample is


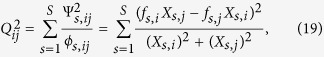


which is independent of *N*_*s*_ as expected. However, 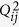 is dependent on *w*_*s*,*i*_, which is consistent with the setup of our model but different from the model in ref. 12. Thus the total sum of the squared errors is


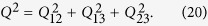


One can see that *a*_*s*_, *b*_*s*_ and *c*_*s*_ could be society-specific and are not included in the final objective function of model calibration.

For each pair of *I*_*c*_ and *t*, a society is excluded in model calibration if the number of agents having at least one collaborator is less than 500 to ensure that *ϵ*_*a*_ has enough realizations. Changing this threshold around 500 results in same results. In addition, if the number of societies included in a model is less than 50, we do not calibrate the model because the model has 10 parameters.

To find the solution to the minimization of *Q*^2^, the taboo search algorithm is adopted^47^. The solution space is restricted to 0 ≤ *γ*_*ij*_ ≤ 2 for 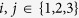 and 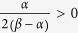. Because there are 10 free parameters, it is not easy to reach the global minimum. We thus perform a taboo search in each cell of a 9-dimensional lattice of size 2^9^ with the constraint that 0 ≤ *γ*_*ij*_ ≤ 1 or 1 ≤ *γ*_*ij*_ ≤ 2. The parameters in certain cell corresponding to the minimum of *Q*^2^ in all cells are obtained as the solution. The normality assumption of fitting errors has been verified by QQ-plots (Fig. S7), which rationalizes the setup of the model. We note that the partitioning of the solution space into a 9-dimensional lattice of size 2^9^ is very important. If we perform the taboo search directly, the resulting *Q*^2^ value is significantly larger and the three preference curves *γ*_*ij*_(*t*) for each *i* are not well separated around *γ*_*ij*_ = 1 (cf. Fig. 2, Fig. S4 and Fig. S5).

### Significance tests

To test whether the preference coefficient *γ*_*ij*_ of *i*-agents to *j*-agents is significantly different from the no-preference case, we perform *F*-tests using the null hypothesis


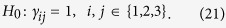


Following ref. 12, the *F*-statistic is


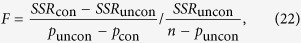


where *SSR* is the sum of squared residuals of the best-fit calibration, *p* is the number of model parameters, *n* is the number of observations, while the subscript “con” indicates the constrained model under the null hypothesis and the subscript “uncon” the unconstrained model.

### Economic output of individuals

There are two virtual currencies, *Xingbi* and *Jinbi*. *Xingbi* cannot be produced by an agent’s activity and can only be bought from the system, which has an approximately stable exchange rate in reference to the Chinese currency *Renminbi*. *Xingbi* is thus a universal currency across different virtual worlds. *Jinbi*, on the other hand, is produced by the economic activities of the agents. There is a built-in exchange platform in each virtual world so that agents can exchange *Xingbi* and *Jinbi*. In this way, there is a real-time exchange rate from *Jinbi* to *Xingbi*.

An agent can produce virtual items (e.g., weapons, clothes, and medicines) and a limited amount of the virtual currency *Jinbi*. We convert the produced items and *Jinbi* to *Xingbi* to obtain the real economic output of each agent on each day. There is a marketplace in each virtual world in which agents can sell their items that are priced in *Xingbi* or *Jinbi*. The price of an item is determined by the average price of all the trades in the marketplace on a given day. Each produced item can thus be measured in *Xingbi*.

## Additional Information

**How to cite this article**: Xie, W.-J. *et al*. Skill complementarity enhances heterophily in collaboration networks. *Sci. Rep*. **6**, 18727; doi: 10.1038/srep18727 (2016).

## Supplementary Material

Supplementary Information
