# Supplementary material for: Skill complementarity enhances heterophily in collaboration networks
Source: Sci Rep. 2016 Jan 8;6:18727. doi: 10.1038/srep18727 (PMC4705466; doi:10.1038/srep18727)
Supplement: Supplementary Information [file srep18727-s1.pdf]

Skill complementarity enhances heterophily in collaboration networks

Wen-Jie Xie[1,2,3], Ming-Xia Li[2,3], Zhi-Qiang Jiang[1,4], Qun-Zhao Tan[5], Boris Podobnik[6,7,8,9,10], Wei-Xing Zhou[1,3,4], & H. Eugene Stanley[6]

November 11, 2015

[1]School of Business, East China University of Science and Technology, Shanghai 200237, China
[2]Postdoctoral Research Station, East China University of Science and Technology, Shanghai 200237, China
[3]Departmenent of Mathematics, East China University of Science and Technology, Shanghai 200237, China
[4]Research Center for Econophysics, East China University of Science and Technology, Shanghai 200237, China
[5]Shanda Games Ltd., 690 Bibo Road, Shanghai 201203, China
[6]Center for Polymer Studies and Department of Physics, Boston University, Boston, MA 02215, USA
[7]Zagreb School of Economics and Management, 10000 Zagreb, Croatia
[8]Luxembourg School of Business, Luxembourg
[9]Faculty of Civil Engineering, University of Rijeka, 51000 Rijeka, Croatia
[10]Faculty of Economics, University of Ljubljana, 1000 Ljubljana, Slovenia

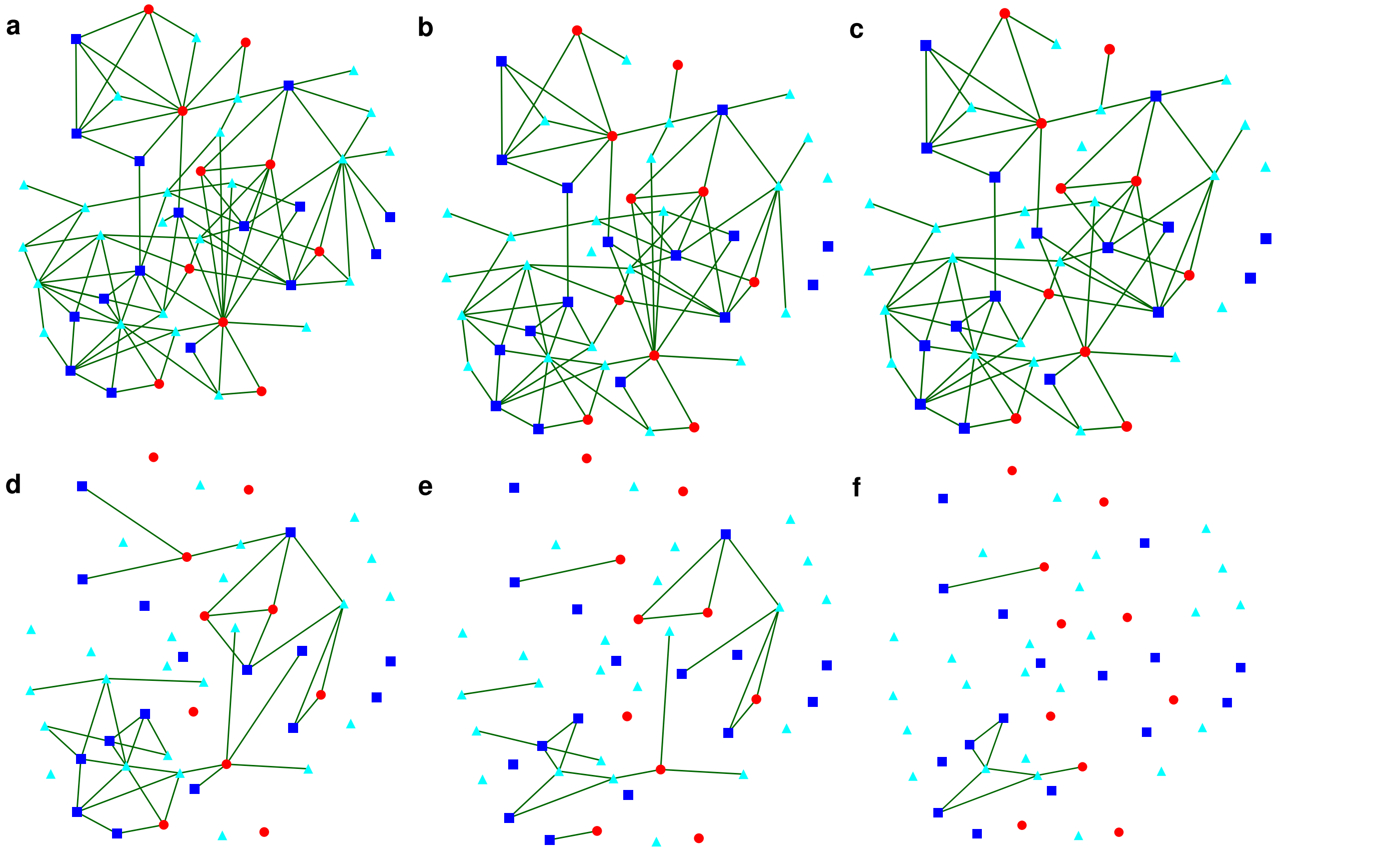

Figure S1: **Examples of collaboration networks.** A small community of 50 agents is chosen to show the structure of the collaboration network in a virtual society with three types of agents (warriors, priests, mages). **a,** The case of $I_c = 0$ in which a link is plotted if the two associated agents are present in their friend list. The network is connected as one "giant component." **b,** $I_c = 1$. **c,** $I_c = 10$. **d,** $I_c = 500$. **e,** $I_c = 1000$. **f,** $I_c = 2000$.

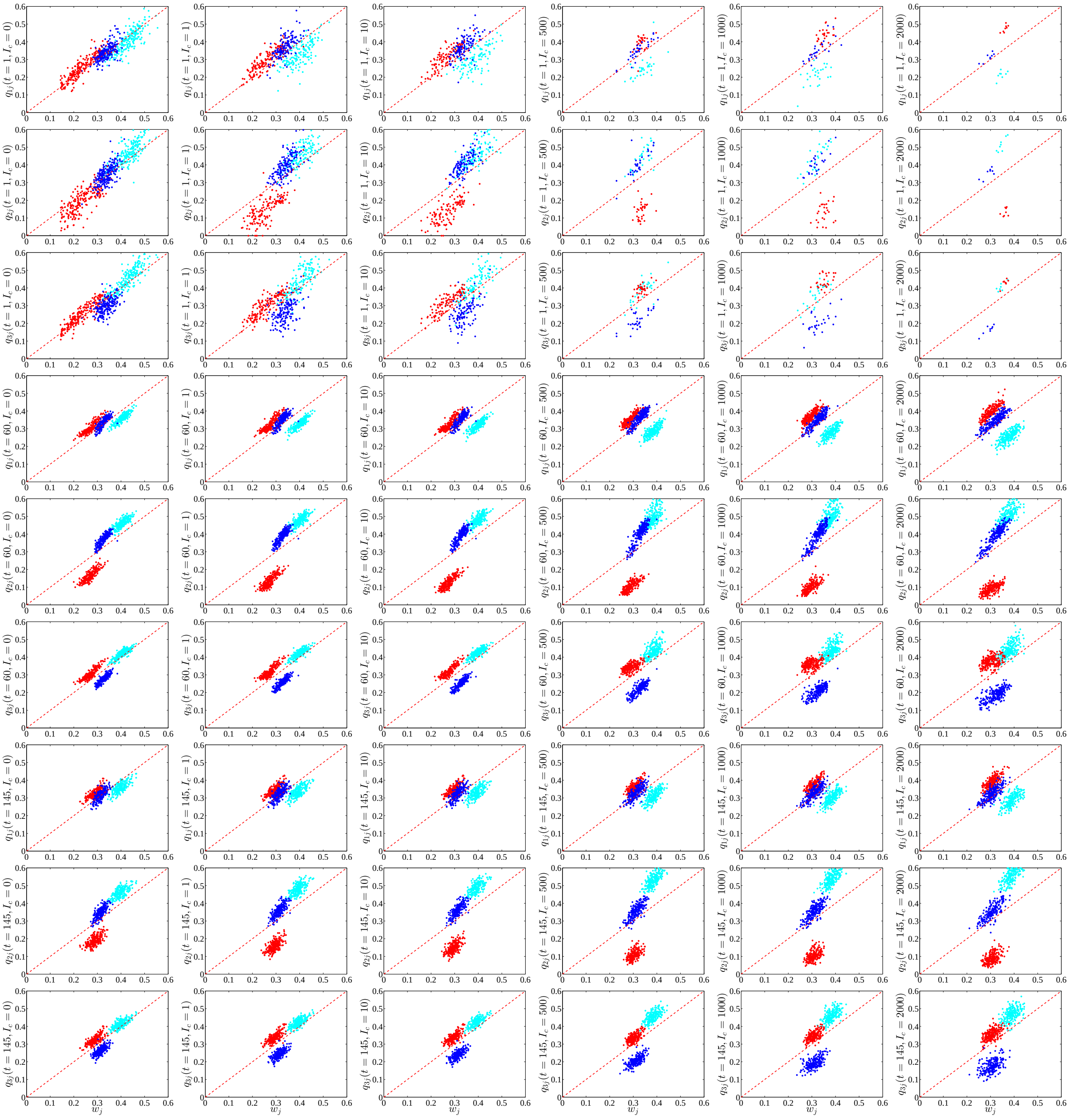

Figure S2: **The average proportion of j-agents in all collaborators of the i-agent q_{ij} versus the ratio w_j of j-agents in the whole collaboration network for $\mathcal{N}_s(I_c, t)$.** The dates are $t = 1$, 60, and 145 and the intimacy thresholds are $I_c = 0, 10, 100, 200, 500$, and 1000 from left to right. In each plot, $j = 1$, 2, and 3 correspond to the three types of agents (warriors, priests, mages).

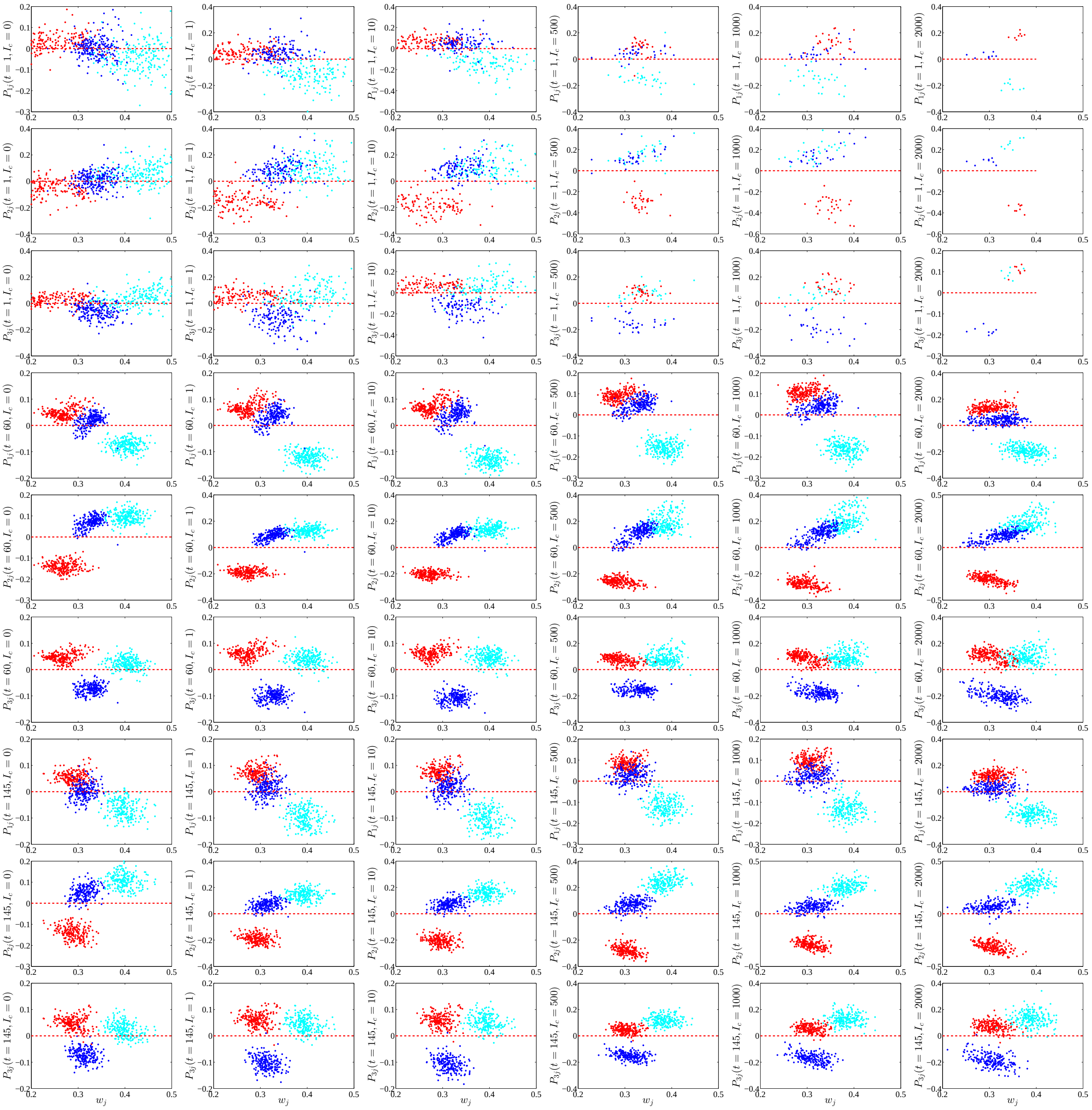

Figure S3: **Preference measure from i-agents to j-agents $P_{ij}(t)$ versus the ratio of j-agents in the whole collaboration network w_j for $\mathcal{N}_s(I_c, t)$.** The dates are $t = 1$, 60, and 145 and the intimacy thresholds are $I_c = 0, 10, 100, 200, 500$, and 1000 from left to right. In each plot, $j = 1$, 2, and 3 correspond to the three types of agents (warriors, priests, mages).

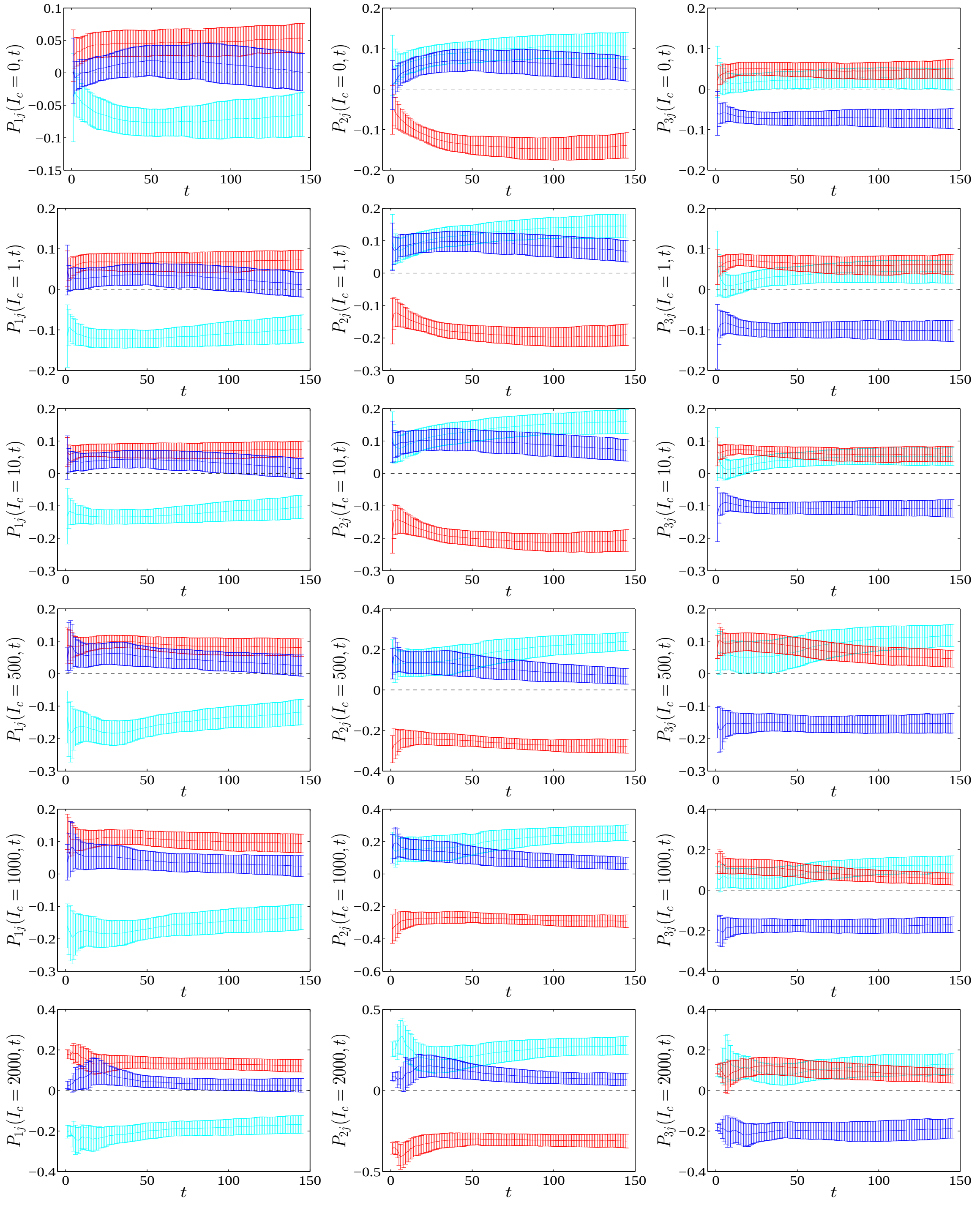

Figure S4: **Evolution of the preference measure $P_{ij}(I_c, t)$ from i-agents to j-agents.** The intimacy thresholds are $I_c = 0$, 1, 10, 500, 1000 and 2000 from top to bottom. In each plot, $j = 1$, 2, and 3 correspond to the three types of agents (warriors, priests, mages).

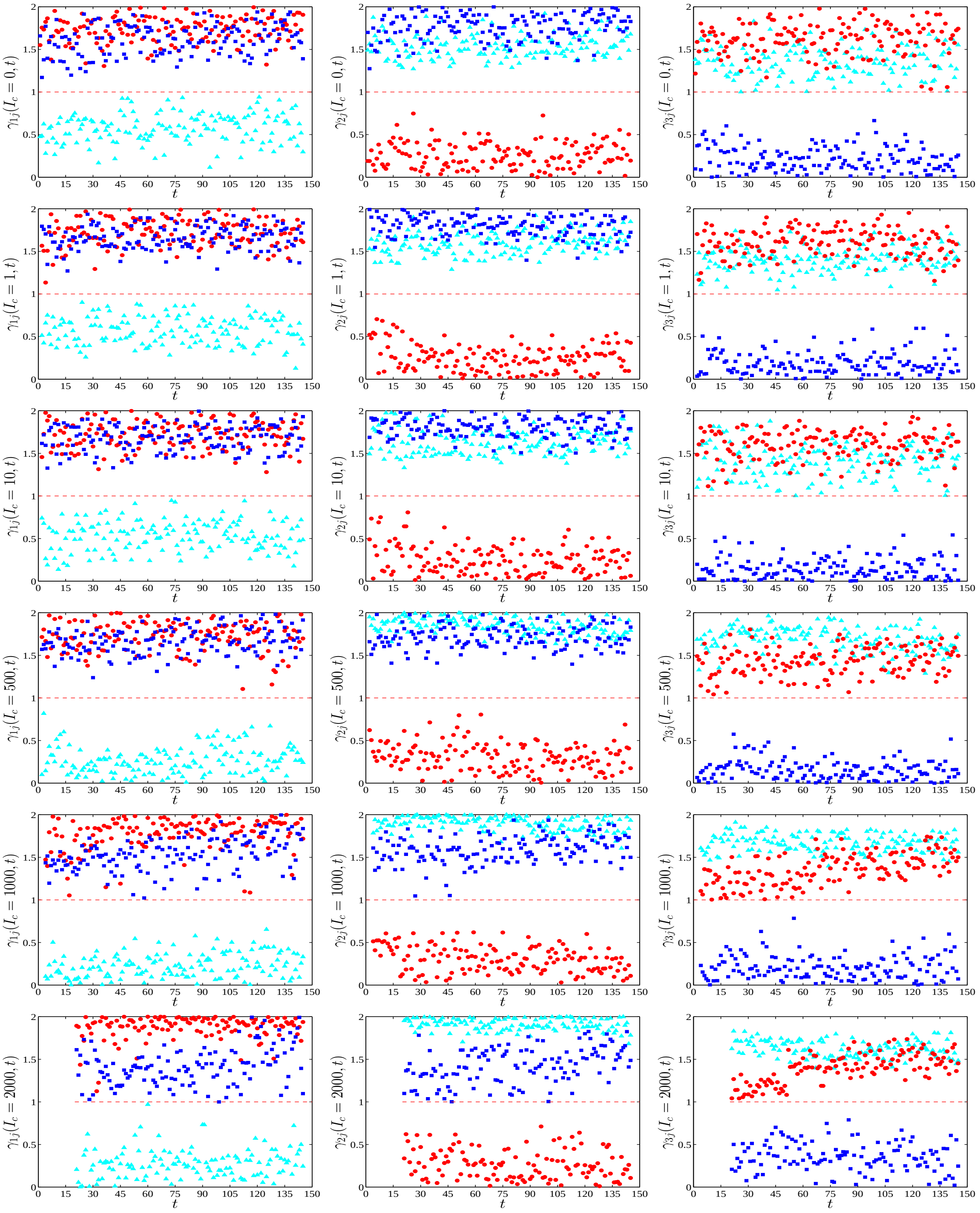

Figure S5: **Evolution of the estimated preference coefficient $\gamma_{ij}(I_c, t)$ from i-agents to j-agents for different thresholds I_c.** Each row corresponds to an intimacy threshold I_c with $I_c = 0, 1, 10, 500, 1000$, and 2000 from top to bottom. In each plot, $j = 1$, 2, and 3 correspond to the three types of agents (warriors, priests, mages). The $\gamma_{ii}(I_c, t)$ coefficients are basically less than 1 with very few exceptions, indicating that the agents do not prefer to collaborate with same-profession agents and are thus heterophilious. The $\gamma_{ij}(I_c, t)$ coefficients with $i \neq j$ are basically greater than 1 with very few exceptions, indicating that the agents prefer to collaborate with different-profession agents. Warriors show comparative preference to priests and mages when I_c is small and prefer to collabotate with priests than mages when I_c is large. Mages prefer more to priests when I_c is small and prefer more to warriors when I_c is large.

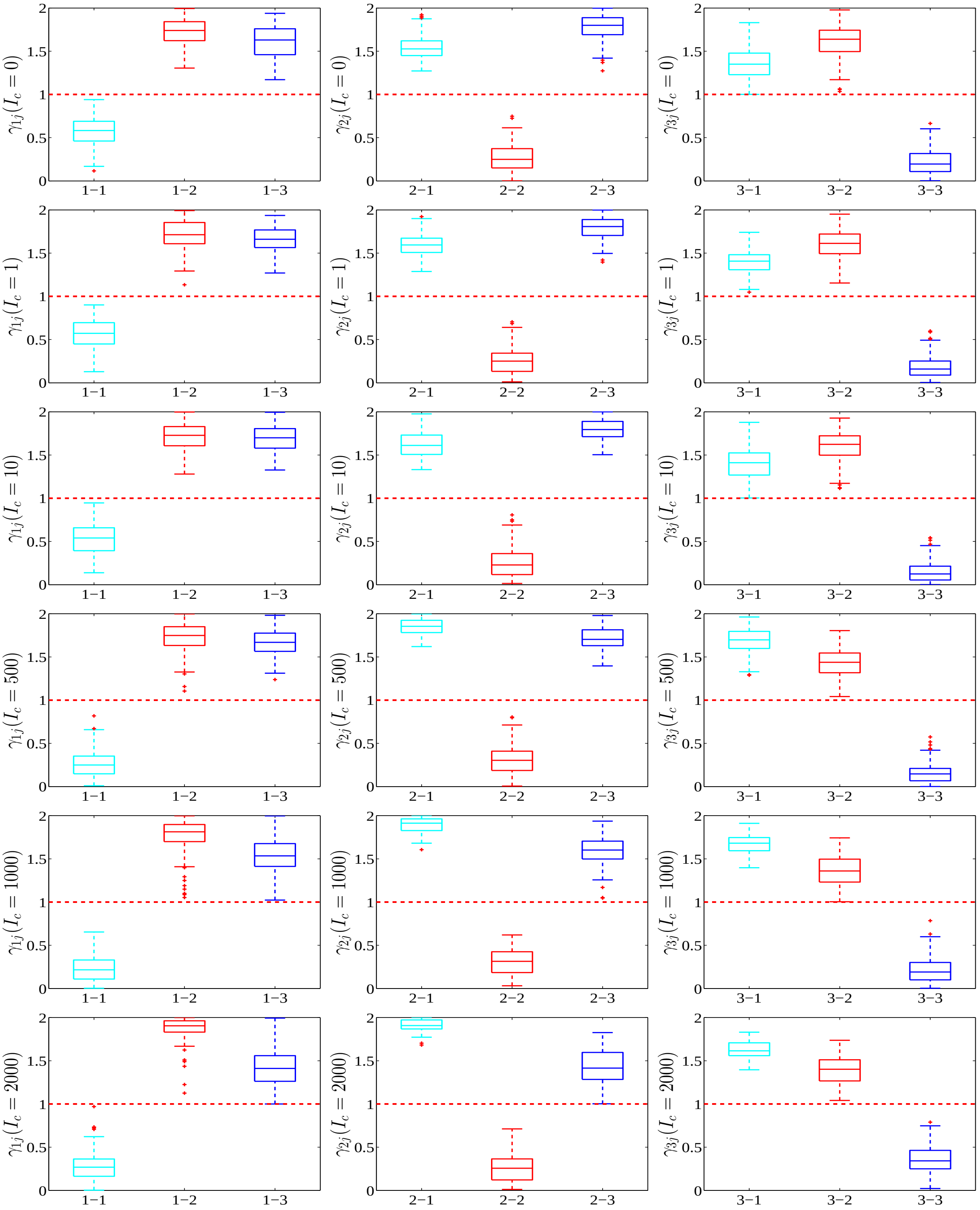

Figure S6: **Evolution of the estimated preference coefficient $\gamma_{ij}(I_c, t)$ from i-agents to j-agents for different thresholds I_c.** Each row corresponds to an intimacy threshold I_c with $I_c = 0, 1, 10, 500, 1000$, and 2000 from top to bottom. In each plot, $j = 1$, 2, and 3 correspond to the three types of agents (warriors, priests, mages).

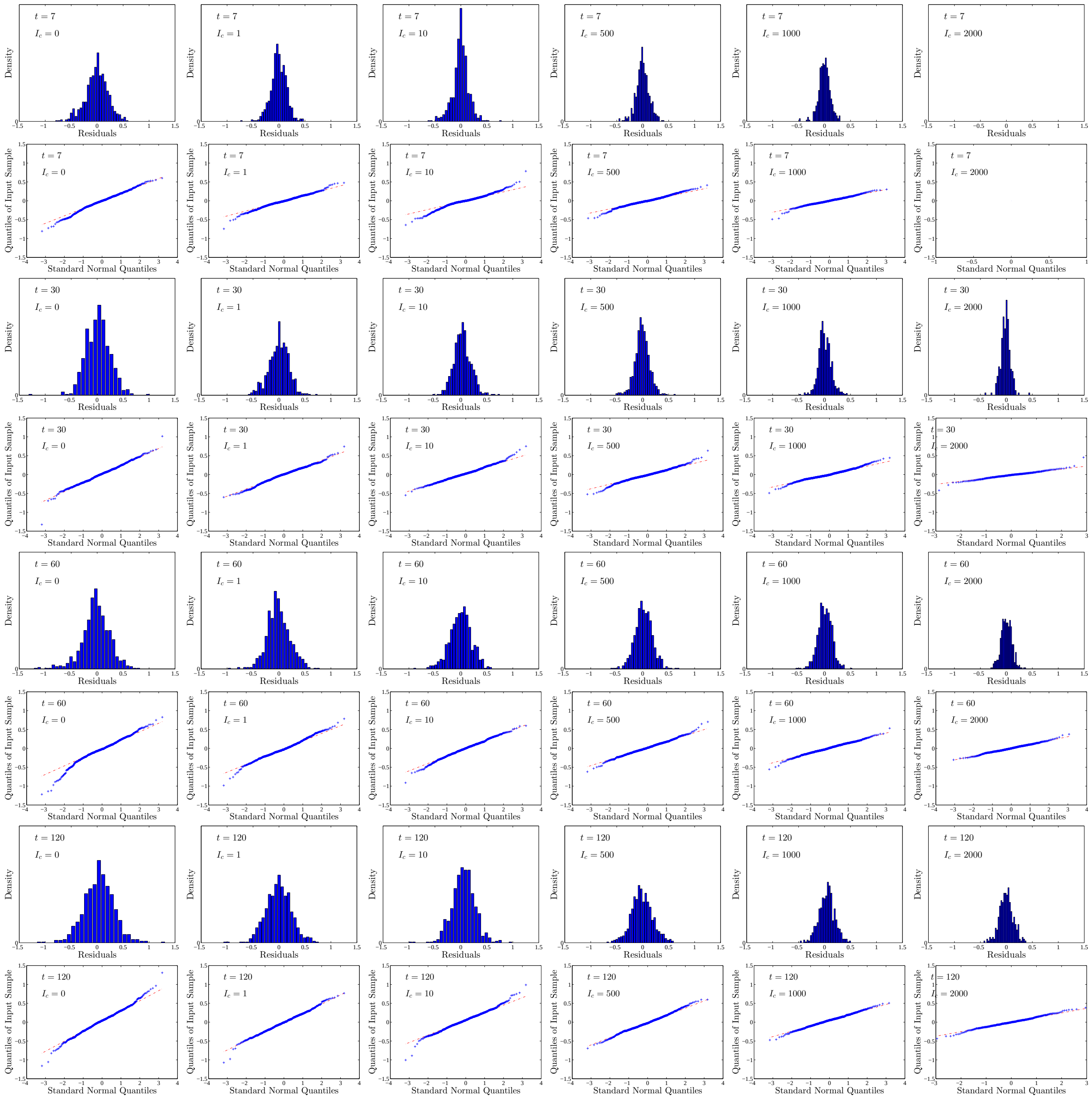

Figure S7: **Testing the Normalty assumption of the error distributions.** The distribution density of the residuals of model calibration and the corresponding QQ-plot are illustrated. It shows that the realized distribution of errors does not differ significantly from the Normal distribution.

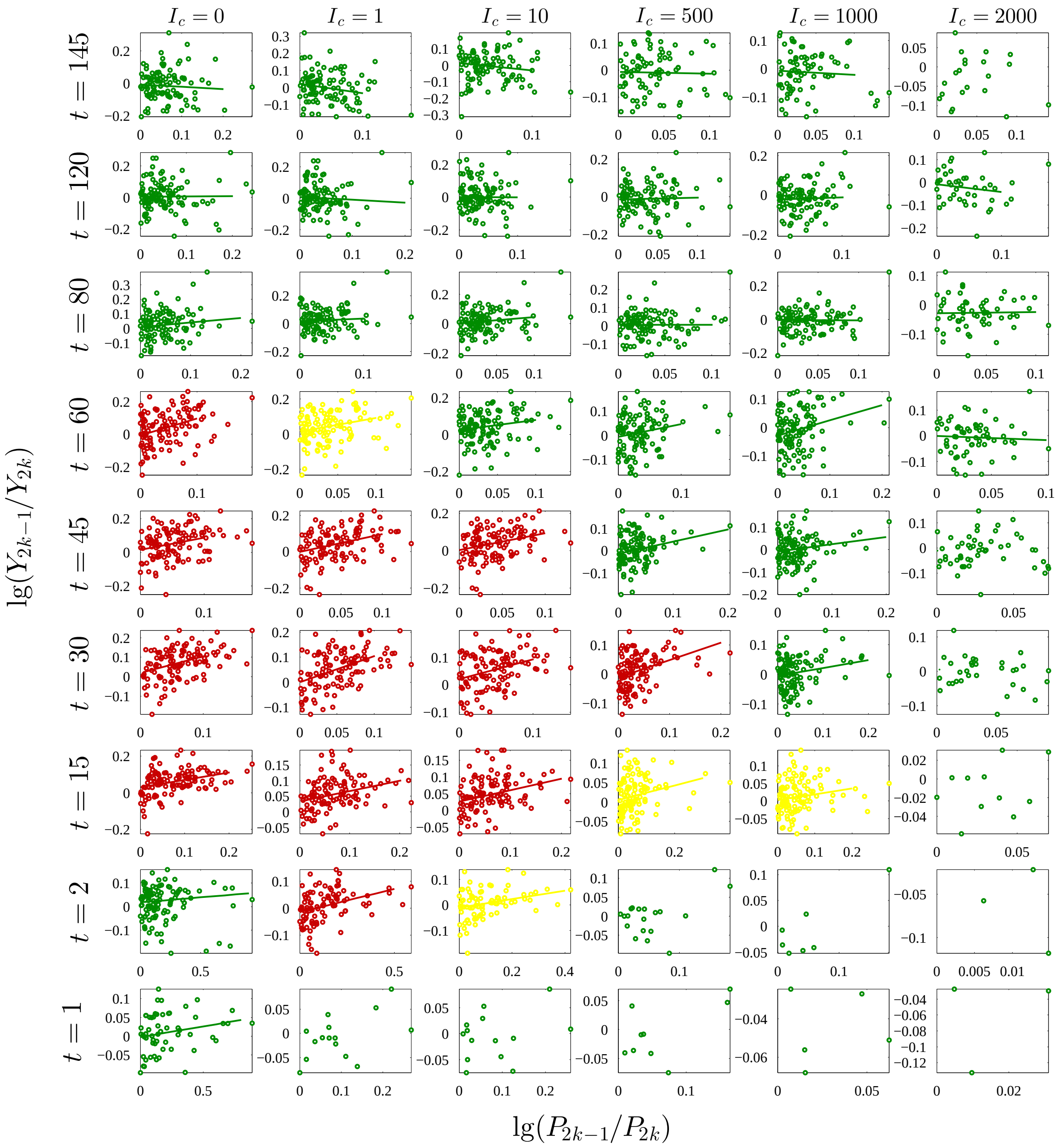

Figure S8: **Relationship between the relative preference $\lg\left(P_{2k-1}/P_{2k}\right)$ and relative economic output $\lg\left(Y_{2k-1}/Y_{2k}\right)$ for different I_c and t.** For each I_c and t, the relative economic yield $\lg(Y_{2k-1}/Y_{2k})$ and the relative preference $\lg(P_{2k-1}/P_{2k})$ are calculated for each society, which is presented as a point in the corresponding plot. The four colors stands for four significance levels (p-values) of the correlation: $p < 0.001$ for **red** plots, $0.001 \leq p < 0.01$ for yellow plots, and $0.01 \leq p$ for **green** plots.

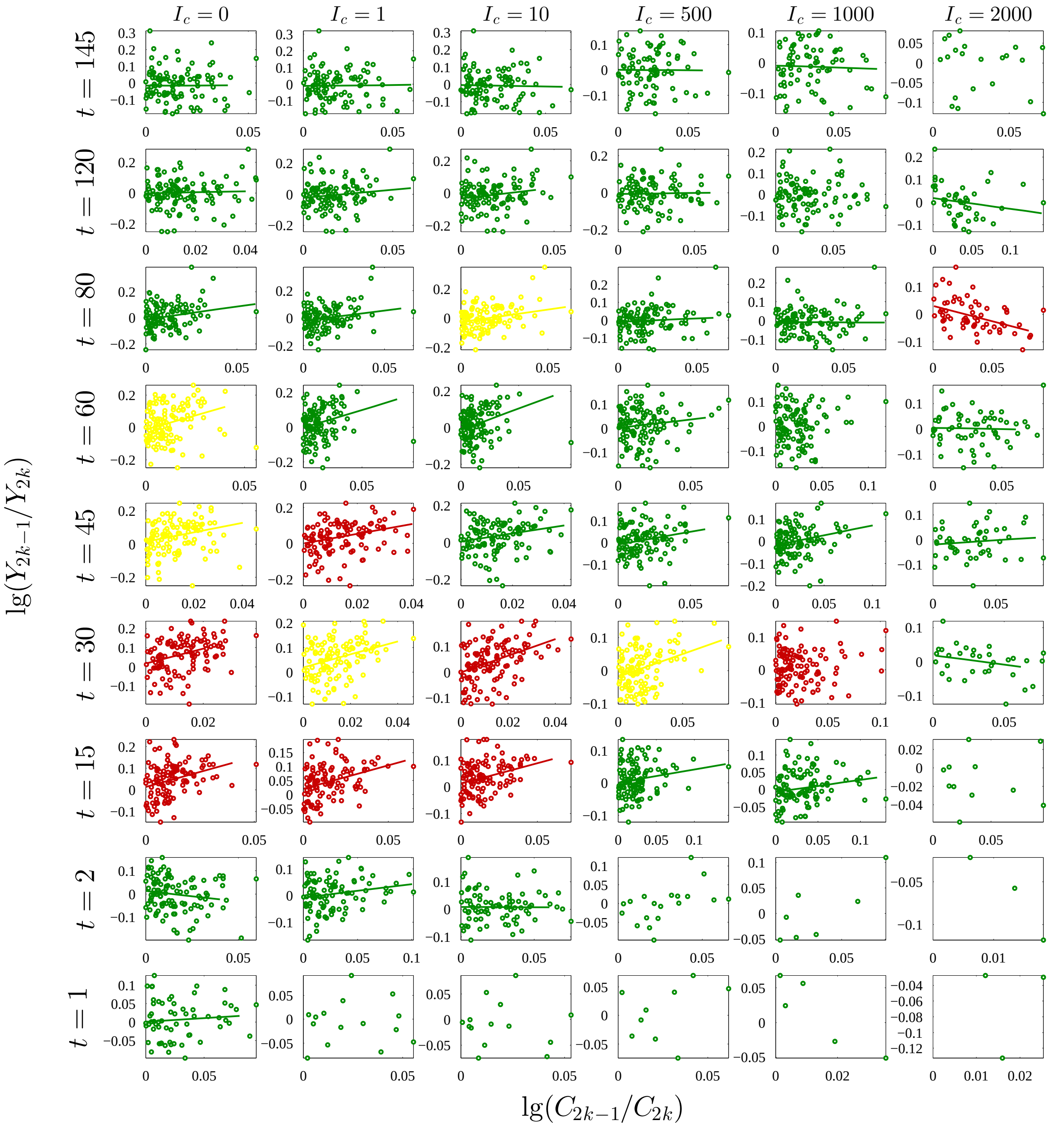

Figure S9: **Relationship between the relative complementarity $\lg(C_{2k-1}/C_{2k})$ and the relative economic output $\lg(Y_{2k-1}/Y_{2k})$ for different I_c and t.** For each I_c and t, the relative economic yield $\lg(Y_{2k-1}/Y_{2k})$ and the relative complementarity $\lg(C_{2k-1}/C_{2k})$ are calculated for each society, which is presented as a point in the corresponding plot. The four colors stands for four significance levels (p-values) of the correlation: $p < 0.001$ for **red** plots, $0.001 \leq p < 0.01$ for **yellow** plots, and $0.01 \leq p$ for **green** plots.
